# Supplementary material for: A small molecule promotes cartilage extracellular matrix generation and inhibits osteoarthritis development
Source: Nat Commun. 2019 Apr 23;10:1914. doi: 10.1038/s41467-019-09839-x (PMC6478911; doi:10.1038/s41467-019-09839-x)
Supplement: Supplementary file 1 — Supplementary Information [file 41467_2019_9839_MOESM1_ESM.pdf]

Supplementary Information

A small molecule promotes cartilage extracellular matrix generation and inhibits osteoarthritis development

Shi et al.

N-[2-bromo-4-(phenylsulfonyl)-3-thienyl]-2-chlorobenzamide (BNTA)

The characterization data of BNTA: **Melting point (mp):** 148-149 °C; **^{1}H-NMR** (600 MHz, DMSO-d_6) δ: 10.15 (s, 1H, N-*H*), 8.68 (s, 1H, thiophene), 7.52-7.94 (m, 9H, aryl); **^{13}C-NMR** (150 MHz, DMSO-d_6) δ: 165.72, 140.97, 137.73, 135.98, 135.56, 134.32, 132.54, 131.88, 130.78, 130.40, 129.98, 129.62, 127.70, 127.59, 115.55; **HRMS**: $[M+H]^+$ calcd for $C_{17}H_{12}NO_3S_2ClBr$, 455.9131; found, 455.9128.

Supplementary figure legends

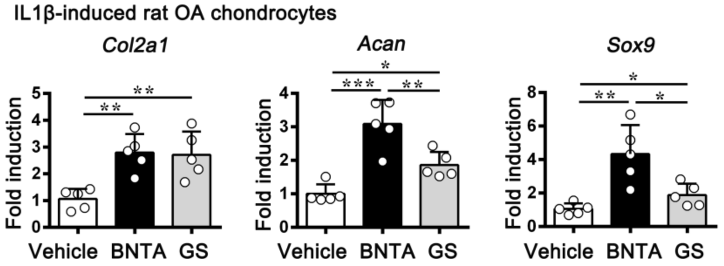

Supplementary Fig. 1 The collagen type II alpha 1 chain (*Col2a1*), aggrecan (*Acan*), and SRY-box 9 (*Sox9*) mRNA levels were evaluated after BNTA (0.1 μM) treatment compared with glucosamine sulfate (GS, 500 μM) in interleukin 1 beta (IL1β, 10 ng ml^{-1})-induced rat osteoarthritis (OA) model at 6 h (n = 5 for each group; *Col2a1, Acan,* one-way ANOVA; *Sox9,* nonparametric test). Data are shown as the mean ± standard deviation (s. d.). $*P < 0.05$, $**P < 0.01$, $***P < 0.001$. Source data are provided as a Source Data file.

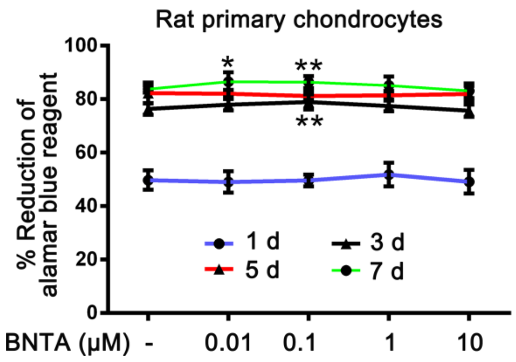

Supplementary Fig. 2 Cell viability of rat primary chondrocytes detected using the alamar blue assay after BNTA treatment for 1 d, 3 d, 5 d, and 7 d (n = 14 for each group; 1 d and 3 d, one-way ANOVA; 5 d and 7 d, nonparametric test). Data are shown as the mean ± standard deviation (s. d.). $*P < 0.05$, $**P < 0.01$. Source data are provided as a Source Data file.

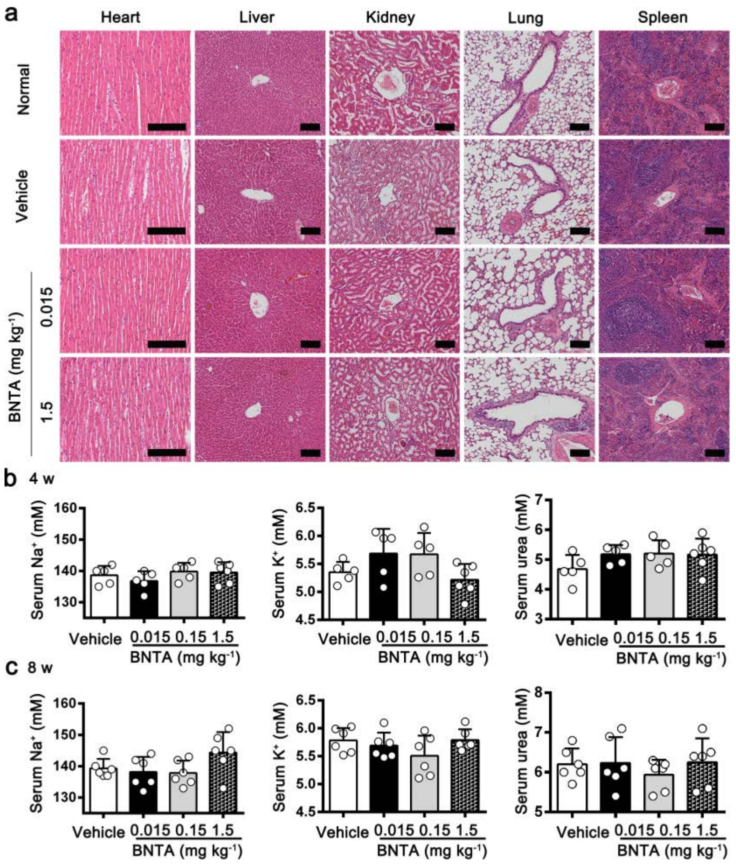

Supplementary Fig. 3 Toxicity of BNTA was detected with H&E staining and biochemical assays of serum *in vivo*. **a**, Histological features of rat heart, liver, kidney, lung, and spleen stained with H&E after treatment with BNTA or vehicle at 8 w (scale bars, 200 μm; n = 4). **b**, Concentrations of serum Na^+, K^+, and urea of rats treated with BNTA or vehicle evaluated by biochemical assays at 4 w (vehicle, 0.015, 0.15 mg kg^{-1}, n = 5; 1.5 mg kg^{-1}, n = 6; one-way ANOVA). **c**, Concentrations of serum Na^+, K^+, and urea of rats in BNTA or vehicle groups at 8 w (n = 6 for each group; one-way ANOVA). Data are shown as the mean ± standard deviation (s. d.). Source data are provided as a Source Data file.

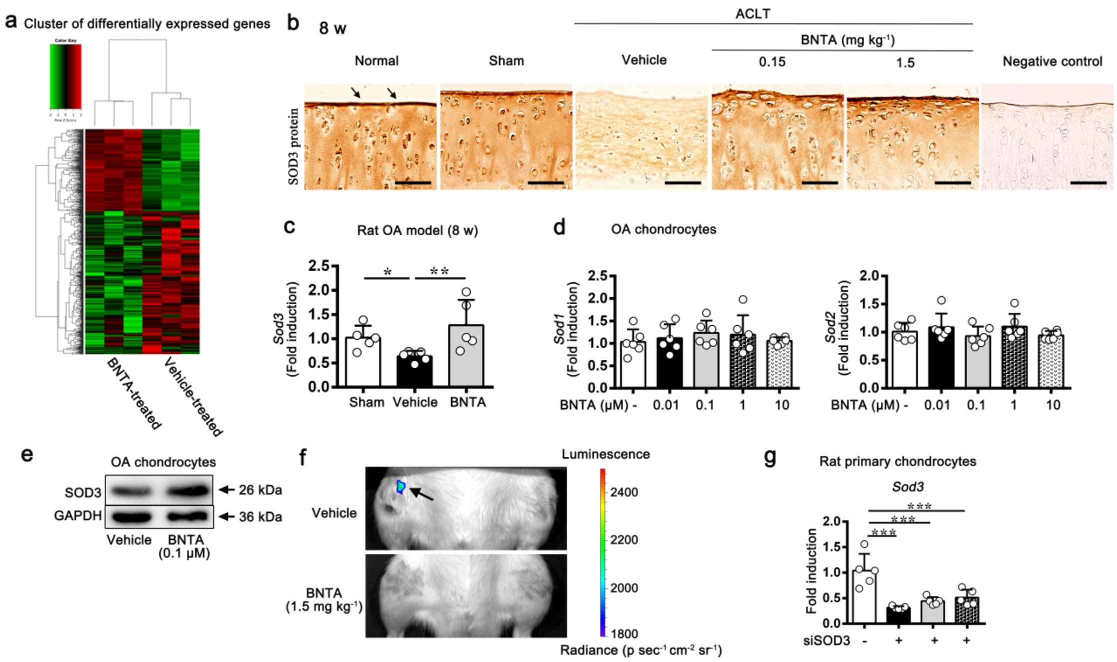

Supplementary Fig. 4 Superoxide dismutase 3 (SOD3) was activated by BNTA. **a**, Cluster of differentially expressed genes in BNTA or vehicle-treated anterior cruciate ligament transection (ACLT) rats assessed by RNA sequencing. **b**, Immunostaining for SOD3 protein in normal, sham, vehicle, and BNTA-treated (0.15, 1.5 mg kg^{-1}) rats at 8 w (brown, arrows; scale bar, 50 μm). **c**, *Sod3* mRNA levels in cartilage tissue of the rat osteoarthritis (OA) model at 8 w (nonparametric test; n = 5 for each group). **d**, Quantification of mRNA levels for *Sod1* and *Sod2* in interleukin 1 beta (IL1β, 10 ng ml^{-1})-induced rat OA chondrocytes after BNTA or vehicle incubation for 6 h (one-way ANOVA; n = 6 for each group). **e**, SOD3 protein levels in IL1β (10 ng ml^{-1})-induced rat OA chondrocytes from the BNTA (0.1 μM) or vehicle groups at 2 d. **f,** Chemiluminescence signal of the ACLT-induced knee joint after vehicle or BNTA (1.5 mg kg^{-1}) treatment at 8 w with intravenous injection of luminol. Arrow showed the reactive oxygen species (ROS) produced in the knee joint. **g**, *Sod3* mRNA levels in rat primary chondrocytes after treatment with three siSOD3s at 24 h (one-way ANOVA; n = 5 for each group). Data are shown as the mean ± standard deviation (s. d.). $*P < 0.05$, $**P < 0.01$. Source data are provided as a Source Data file.

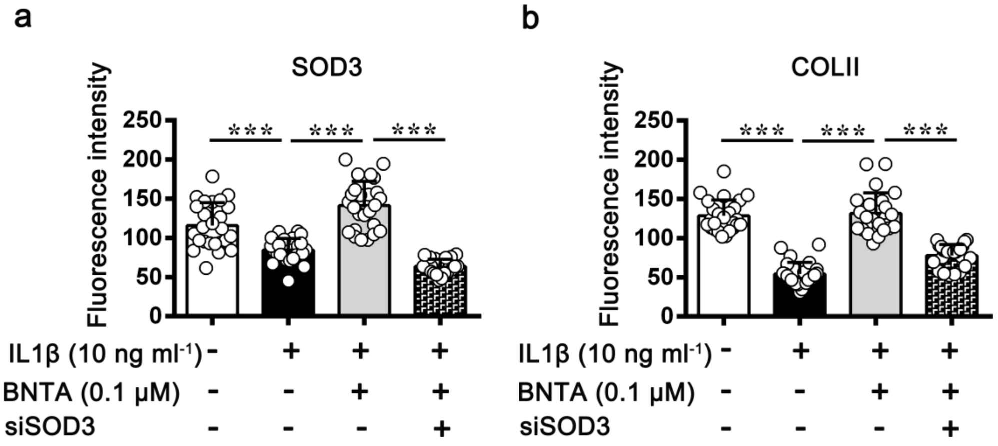

Supplementary Fig. 5 The fluorescence intensity of superoxide dismutase 3 (SOD3) and type II collagen (COLII) in rat primary chondrocytes was quantified using LAS_X software when cultured with interleukin 1 beta (IL1β, 10 ng ml^{-1}), BNTA (0.1 μM), or siSOD3 for 2 d (n = 25 for each group; nonparametric tests). Data are shown as the mean ± standard deviation (s. d.). ***P < 0.001. Source data are provided as a Source Data file.

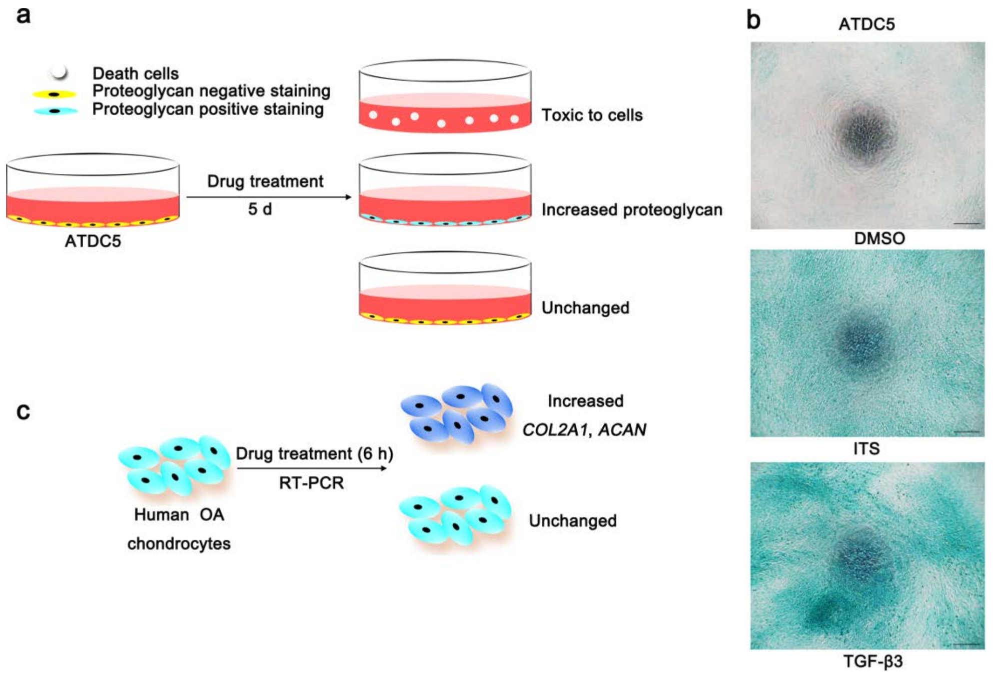

Supplementary Fig. 6 Development of an effective assay to screen for candidate of disease-modifying osteoarthritis drug (DMOAD). **a**, Scheme of alcian blue staining outcomes after treating ATDC5 cells with the small compounds library for 5 d. **b**, Alcian blue staining in ATDC5 cells after stimulation with transforming growth factor-β3 (TGF-β3) or insulin-transferrin-selenium (ITS; scale bar, 200 μm). **c**, Schematic diagram of possible results after treating human osteoarthritis (OA) chondrocytes with prospective candidates, as evaluated with reverse transcription polymerase chain reaction (RT-PCR).

Supplementary Table 1 The excluded candidates for the experiments

Product Name	References
NS-398	*J RHEUMATOL*. 28, 2509-2519 (2001)
Melatonin	*Br J Pharmacol*. 175, 3230–3238 (2018)
Harmine HCl	*BIOCHIMIE*. 95, 374-381 (2013)
Kaempferol	*Med Sci Monit*. 23, 3925-3931 (2017)
CL-82198	*CURR MED CHEM*. 18, 977-1001 (2011)
Z-FA-FMK	*OSTEOARTHR CARTILAGE*. 23, A57 (2015)
SB220025	*SCAND J RHEUMATOL*. 36, 247-258 (2007)
SD-208	*GROWTH FACTORS*. 24, 268-278 (2006)
Cryptotanshinone	*INT IMMUNOPHARMACOL*. 50, 161-167 (2017)
CXCR4 Antagonist I, AMD3100	*J ORTHOP RES*. 33, 1071-1078 (2015)
SB 203580, Sulfone	*McGill University Montreal,(Quebec),Canada*. (2011)
Histone Deacetylase Inhibitor	*Osteoarthritis Cartilage*. 21, 1987-1996 (2013).
CP-690550	*JAMA*. 297, 28-29 (2007)
Propyl gallate	*Free Radic Res*. 30, 395-405 (1999)
Sphingosine	*Osteoarthritis Cartilage*. 6, 393-399 (1998)
Kenpaullone	*INT J CLIN EXP MED*. 4, (2016)
Pifithrin-α, Cyclic-	*Arthritis Rheum*. 60, 2340-2349 (2009)
SIRT1 Inhibitor IV, (S)-35	*J ORTHOP RES*. 29, 511-515 (2011)
SAHA	*AM J PATHOL*. 186, 2701-2708 (2016)
Prostaglandin J2	*J BIOL CHEM*. 279, 37939-37950 (2004)
5-Aza-2-deoxycytidine	*ANN RHEUM DIS*. 66, 1616-1621 (2007)
Amphotericin B	*INFECTION*. 25, 112-116 (1997)
JNK Inhibitor	*Arthritis Rheum*. 56, 2663-2673 (2007)
Oxytetracycline, a -apo-	*J BONE MINER METAB*. 28, 627-633 (2010)
p38 MAP Kinase Inhibitor VI, JX401	*J Inflamm (Lond)*. 5, 22 (2008)
p38 MAP Kinase Inhibitor VIII	*J Inflamm (Lond)*. 5, 22 (2008)
E-caryophyllene	*EUR J PHARMACOL*. 750, 141-150 (2015)
Alsterpaullone	*MOL MED REP*. 7, 603-607 (2013)
Carnosic acid	*MOLECULES*. 21, 465 (2016)
PKCbII/EGFR Inhibitor	*Mediators Inflamm*. 2013, 326041 (2013)
Lavendustin A	*Proc Natl Acad Sci U S A*. 105, 7467-7471 (2008)
Tranylcypromine	*ARTHRITIS RES THER*. 16, R113 (2014)
JAK Inhibitor I	*Tissue Eng Part A*. 20, 2243-2252 (2014)
CCR2 Antagonist	*ANN RHEUM DIS*. 76, 914-922 (2017)
A77 1726	*ARTHRITIS RES THER*. 6, R181-R189 (2004)
trans-Retinoic Acid	*J CELL PHYSIOL*. 159, 340-346 (1994)
Cyclopamine	*INT J MOL SCI*. 14, 5966-5977 (2013)

Adenosine	*Osteoarthritis Cartilage*. 14, 486-495 (2006)
Gingerol	*J CELL BIOCHEM*. 118, 1003-1013 (2017)
Lycorine HCl	*MOL MED REP*. 14, 3389-3396 (2016)
Nicotine, (-)-	*Osteoarthritis Cartilage*. 13, 942-943 (2005)
URB-597	*PAIN*. 152, 975-981 (2011)
Bisindolylmaleimide I	*J BIOL CHEM*. 282, 2929-2936 (2007)
Gö 6976	*J BIOL CHEM*. 281, 24124-24137 (2006)
NDGA	*Arthritis Rheum*. 26, 771-774 (1983)
Tetramethylpyrazine	*J ETHNOPHARMACOL*. 132, 414-420 (2010)
Mithramycin A	*ARTHRITIS RES THER*. 7, R777-R783 (2005)
Actinonin	*MATRIX BIOL*. 19, 333-344 (2000)
GM6001	*Osteoarthritis Cartilage*. 14, 738-748 (2006)
Enalapril	*DARU: Journal of Pharmaceutical Sciences*. 15, 199-204 (2007)
SB 203580	*J IMMUNOL*. 161, 467-473 (1998)
SB202190	*Osteoarthritis Cartilage*. 18, 1509-1517 (2010)
BMS-5	*PLOS ONE*. 6, e28663 (2011)
MCI-186	*Biochem Biophys Res Commun*. 473, 840-844 (2016)
EPA	*ARTHRITIS RES THER*. 12, R207 (2010)
Thymoquinone	*xp Biol Med (Maywood)*. 235, 1425-1431 (2010)
Tyrphostin 1	*Arthritis Rheum*. 48, 404-409 (2003)
Tetrandrine	*EVID-BASED COMPL ALT*. 2013, 1-8 (2013)
Quercetin	*J NUTR BIOCHEM*. 23, 106-112 (2012)
Myricetin	*INT J PHARMACOL*. 4, 440-450 (2016)
Cambinol	*JOINT BONE SPINE*. 79, 570-573 (2012)
17β-Estradiol	*BONE*. 39, 310-317 (2006)
Splitomycin	*JOINT BONE SPINE*. 79, 570-573 (2012)
Salermide	*JOINT BONE SPINE*. 79, 570-573 (2012)
Baicalein	*CELL PHYSIOL BIOCHEM*. 36, 325-333 (2015)
Betulinic Acid	*INT IMMUNOPHARMACOL*. 29, 687-692 (2015)
Phorbol 12,13-dibutyrate	*EXP CELL RES*. 201, 245-249 (1992)
